# 2438. Positive Cerebrospinal Fluid Cultures from Intraventricular Reservoirs for CLN2 Patients: One Institution's Experience

**DOI:** 10.1093/ofid/ofad500.2057

**Published:** 2023-11-27

**Authors:** Gianna Fote, Amanda Schafenacker, Daniel C Sherlock, Justin So, Alexander Himstead, Jordan Davies, Sarah Pedroza, Wendi Gornick, Raymond Wang, Joffre Olaya, Antonio C Arrieta, Jasjit Singh

**Affiliations:** University of California, Irvine, Orange, California; UC Irvine / Children's Hospital Orange County, Orange, California; Children's Hospital of Orange County, Newport Beach, California; University of California, Irvine, Orange, California; University of California at Irvine, Orange, California; Children's Hospital of Orange County, Newport Beach, California; Children's Hospital Orange County, Orange, California; Children's Hospital Orange County, Orange, California; Children's Hospital of Orange County, Newport Beach, California; Children's Hospital of Orange County, Newport Beach, California; Children's Hospital of Orange County, Orange, California, USA, Orange, California; CHOC Children's Hospital, Orange, California

## Abstract

**Background:**

Enzyme replacement therapy (ERT) with cerliponase alfa (Brineura) has been shown to slow progression of milestone deterioration in Neuronal Ceroid Lipofuscinosis Type 2 (CLN2), an inherited neurodegenerative lysosomal storage disorder. Brineura must be administered intraventricularly every 2 weeks, necessitating the placement of ventricular reservoirs and requiring frequent access. Traditionally, positive cerebrospinal fluid (CSF) cultures are managed with device replacement and antibiotic treatment. We sought to establish which circumstances might allow for careful observation without device removal. In this study, we report our single-institution experience with clinical outcomes of positive CSF cultures in 16 CLN2 patients over 6 years.

**Methods:**

We retrospectively reviewed a cohort of 16 patients with CLN2 disease who had ventricular reservoirs placed for ERT administration. At each ERT infusion, CSF was collected by sterile technique and cultured by both thioglycolate broth and agar plate. Epidemiologic and microbiologic data, symptomatology, total antibiotic days, removal and replacement of ventricular reservoir, hospital length of stay, and mortality were analyzed for each patient.

**Results:**

In our cohort, 11 of 16 patients (69%) had at least one positive CSF culture (Fig 1). Of the 11 with positive cultures, only 3 had their device removed and replaced for a positive culture with concurrent antibiotic treatment at our center, and 2 patients subsequently have been treated with prophylactic antibiotics at infusions (Fig 2). Out of 1401 total CSF cultures, 64 (4.56%) were positive. The most common organism grown was *Cutibacterium acnes*, which typically grew in only broth culture (82%), suggesting low bacterial burden (Fig 3). The other 7 patients with positive cultures remained asymptomatic with no intervention required.

**Figure:**
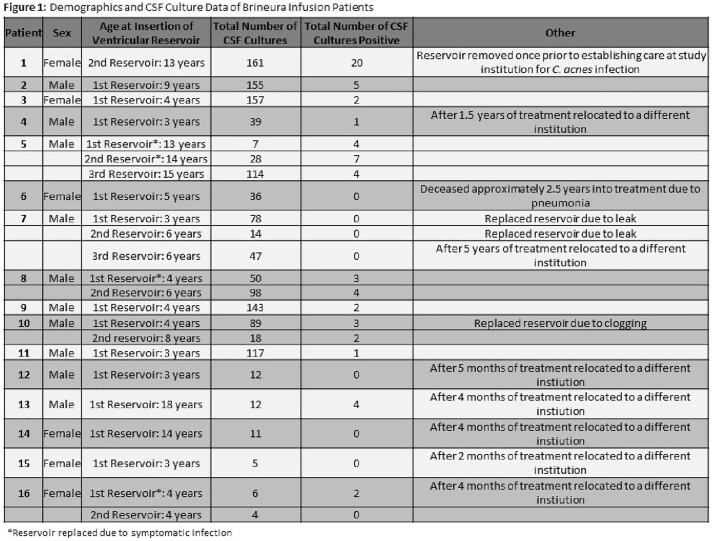

**Figure:**
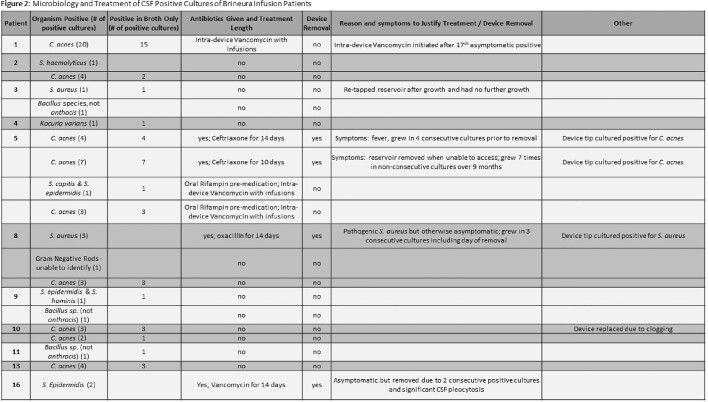

**Figure:**
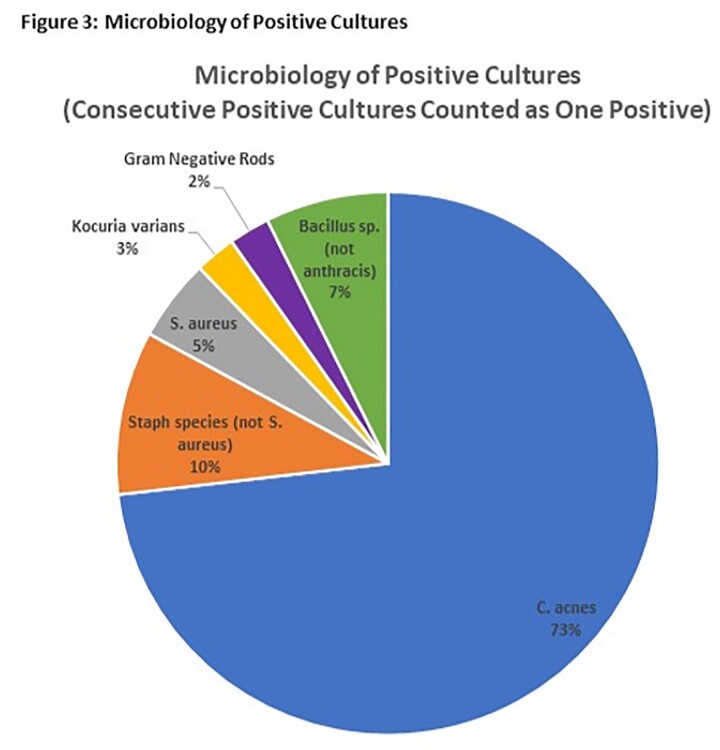

**Conclusion:**

At our institution, we have found that in asymptomatic patients with frequently accessed ventricular reservoirs, positive CSF culture with low virulence skin flora was common, and rarely required intervention. This strategy reduced the risks of invasive surgery, prolonged antibiotic courses, and missed infusions. In the future, routine screening cultures in asymptomatic patients can be avoided.

**Disclosures:**

**Raymond Wang, MD**, Biomarin Pharmaceutical: Advisor/Consultant|Biomarin Pharmaceutical: Grant/Research Support|Biomarin Pharmaceutical: equity interest **Antonio C. Arrieta, MD, FIDSA, FPIDS**, Astellas Pharma Global Development, Inc.: Advisor/Consultant|Astellas Pharma Global Development, Inc.: Grant/Research Support|Astellas Pharma Global Development, Inc.: Honoraria|Cumberland Pharmaceutical: Grant/Research Support|IDbyDNA: Advisor/Consultant|IDbyDNA: Grant/Research Support|Melinta: Grant/Research Support|Merck: Advisor/Consultant|Merck: Grant/Research Support|Nabriva: Grant/Research Support|Paratek Pharmaceuticals: Grant/Research Support|Pfizer, Inc: Advisor/Consultant|Pfizer, Inc: Grant/Research Support|Roche/Genentech: Grant/Research Support|The Medicine Company: Grant/Research Support

